# Engaging media in communicating research on sexual and reproductive health and rights in sub-Saharan Africa: experiences and lessons learned

**DOI:** 10.1186/1478-4505-9-S1-S7

**Published:** 2011-06-16

**Authors:** Rose Ndakala Oronje, Chi-Chi Undie, Eliya Msiyaphazi Zulu, Joanna Crichton

**Affiliations:** 1 Institute of Development Studies, at the University of Sussex, Brighton, BN1 9RE, UK; 2 Population Council, General Accident House, Ralph Bunche Road, Nairobi, Kenya; 3 African Institute for Development Policy (AFIDEP), P. O. Box 14688-00800, Westlands, Nairobi, Kenya; 4 School of Social and Community Medicine, University of Bristol, Canynge Hall, 39 Whatley Road, Bristol, BS8 2PS, UK

## Abstract

**Background:**

The mass media have excellent potential to promote good sexual and reproductive health outcomes, but around the world, media often fail to prioritize sexual and reproductive health and rights issues or report them in an accurate manner. In sub-Saharan Africa media coverage of reproductive health issues is poor due to the weak capacity and motivation for reporting these issues by media practitioners. This paper describes the experiences of the African Population and Health Research Center and its partners in cultivating the interest and building the capacity of the media in evidence-based reporting of reproductive health issues in sub-Saharan Africa.

**Methods:**

The paper utilizes a case study approach based primarily on the personal experiences and reflections of the authors (who played a central role in developing and implementing the Center’s communication and policy engagement strategies), a survey that the Center carried out with science journalists in Kenya, and literature review.

**Results:**

The African Population and Health Research Center’s media strategy evolved over the years, moving beyond conventional ways of communicating research through the media via news releases and newspaper stories, to varying approaches that sought to inspire and build the capacity of journalists to do evidence-based reporting of reproductive health issues. Specifically, the approach included 1) enhancing journalists’ interest in and motivation for reporting on reproductive health issues through training and competitive grants for outstanding reporting ; 2) building the capacity of journalists to report reproductive health research and the capacity of reproductive health researchers to communicate their research to media through training for both parties and providing technical assistance to journalists in obtaining and interpreting evidence; and 3) establishing and maintaining trust and mutual relationships between journalists and researchers through regular informal meetings between journalists and researchers, organizing field visits for journalists, and building formal partnerships with professional media associations and individual journalists.

**Conclusion:**

Our experiences and reflections, and the experiences of others reviewed in this paper, indicate that a sustained mix of strategies that motivate, strengthen capacity of, and build relationships between journalists and researchers can be effective in enhancing quality and quantity of media coverage of research.

## Background

Given their ability to disseminate information in a broad, timely, and accessible manner, the mass media constitute an important source of information for the general public and policymakers. As information providers, the mass media inform, educate, entertain, persuade, socialize, and market commercial products, among other roles [1,2]. Nwokeafor and Nwankwo [3] indicate that mass media communication, as an agent of socialization, disseminates values and information to society. More importantly, the mass media have been shown to have the power to focus public attention on important issues – the media’s agenda-setting role [4]. The recognition of these roles and the critical value of the media in communication is the driving force behind the emphasis of the African Population and Health Research Center (APHRC) in motivating and building the capacity of the mass media to communicate research on various health and socio-development issues, including sexual and reproductive health and rights (SRHR).

APHRC, a research institution based in Nairobi, Kenya, is committed to promoting the use of its study findings in policy and practice, through extensive, focused and sustained dissemination and engagement with key audiences. APHRC conducts research in four broad areas, namely, urbanization and wellbeing, population dynamics and reproductive health, health systems and challenges, and education (see Additional file 1 for a brief summary of APHRC). However, as with many research organizations, the pathways to moving research into policy and action are a major challenge and the Center endeavors to circumvent these challenges to ensure that its research effectively informs policy and program discourses on population and health issues around the continent. The media, which is the focus of this paper, is just one component of APHRC’s approach in influencing policy and practice with research; other components include one-on-one meetings, partnerships with policymakers, participation in relevant government technical working groups, scientific publications, policy briefs, seminars and conferences.

The mass media, as our experience has shown, are not always necessarily interested in nor trained to report on health research. Indeed, media coverage of health has been found to be ‘shallow and reactive, dominated by announcements of new drugs or official health promotion campaigns, and lacking in investigative depth’ [5]. Besides lack of interest, the media in sub-Saharan Africa often lacks motivation, skills and capacity to understand, interpret, and report research findings on health, including SRHR [5]. But the media’s lack of capacity and interest is not the only problem; researchers also often lack the capacity to simplify their research and present it in a way that captures media interest, and they tend to shy away from media fearing misrepresentation and sensationalization of their work [5]. To respond to these dual challenges, APHRC adopted the partnership approach which establishes relationships between individuals, organizations, and institutions [6]. A partnership is associated with long-term shared commitment, responsibility, reciprocal obligation, equality and balance of power [6].

Poor sexual and reproductive health is a persistent and major problem in developing countries. According to UNFPA [7], illnesses and deaths from poor reproductive health account for one-fifth of the global burden of disease. In Africa, only 20% of married women use modern contraception [7]. Of all the regions of the world, Africa has the highest prevalence of deaths due to unsafe abortion [8]. Further, overall maternal mortality rates are highest in the sub-Saharan African region [7].

In Kenya, poor reproductive health is manifested in the high number of deaths and illnesses resulting from HIV/AIDS, childbirth, and abortion. HIV/AIDS kills 140,000 Kenyans every year [9]; of every 100,000 Kenyan women giving birth, 488 die from complications associated with childbirth [10]; and 20,000 Kenyan women are hospitalized every year with abortion-related complications [11]. Other indicators of poor reproductive health in Kenya include adolescents’ lack of access to reproductive health information and services [10], continued female genital mutilation despite the banning of this practice in 2001 through the Children’s Act [10], and low use of contraception [10].

APHRC’s action oriented research program seeks to raise the profile of SRHR issues and provide evidence to guide policies on how to address the challenges. Among the SRHR issues that the Center tackles are: understanding drivers and consequences of population change in Africa, sexuality, gender-based violence; interventions for addressing barriers to contraceptive use, family planning needs of people living with HIV, and strategies for enhancing access to broader SRH services in marginalized communities such as urban slum settlements.

The mass media can contribute greatly to efforts to address these challenges by focusing public attention on these issues, making them more visible in development discourses through their agenda-setting role, and providing accurate and comprehensive information on these issues to the public. In so doing, the media can help break the culture of silence and high levels of stigma and discrimination associated with SRHR issues by promoting openness and public discussions of these issues [12].

This paper discusses APHRC’s experience in working with the media to promote reporting on health research in general, and its own research, in particular over the period 2004 and 2009. The media has the capacity to reach and influence the key target audiences that the Center’s research targets including policymakers, program implementers, key stakeholders, and the public. APHRC’s engagement with the media has varied in its approach, nuanced in its successes, and provides a rich case study to learn from. In the paper, we describe the approaches used and highlight what worked well, the challenges we faced, and overall lessons learnt in order to provide a learning platform for institutions seeking to adopt similar strategies.

## Methodology

The paper utilizes a case study approach that reviews the experiences of APHRC in working with the media to promote evidence-based reporting of SRHR issues and literature review. The case study is based primarily on the personal experiences and reflections of the authors (who played a central role in developing and implementing APHRC’s communication and policy engagement strategies), and a survey that APHRC carried out with science journalists in Kenya. The case study approach was adopted because little is known about the modalities of engaging the media with research, especially in Africa, and the case study approach has been noted as being particularly appropriate for researching an area where few studies have been carried out [13]. APHRC has been working with the mass media since 2004, and the four authors of this paper played key roles in the development and implementation of the Center’s research communications and policy engagement strategy, including media engagement between 2004 and 2009. The team comprised communication specialists and researchers committed to translating research into policy and action. The authors’ personal experiences and reflections offer valuable insights on the complexities of the processes and the lessons learnt.

In addition to the authors’ experiences and reflections from the media engagement activities implemented by APHRC, we also utilized findings from a self-administered survey of eighteen Kenyan science journalists carried out by APHRC in order to understand the key challenges that journalists face in evidence-based reporting of health issues and their insights on how the challenges could be addressed. The respondents included both writers and editors, and the data collection instrument comprised a mix of structured and unstructured questions. The survey was conducted in 2007 by the Media for Environment, Science, Health and Agriculture in Kenya (MESHA) on behalf of APHRC.

Finally, the review of literature enabled us to highlight the role of the mass media in development, including promoting better health. It also enabled us to situate the partnership approach taken by APHRC in the wider literature on the partnership model.

## Results and discussion

### Strategies for engaging the mass media – what has worked and why

In developing media strategy, APHRC identified the need to go beyond the more conventional approaches to media engagement, such as circulating news releases, writing newspaper stories, and holding press conferences, in order to address the challenges of low motivation and weak capacity among targeted practitioners in the media. APHRC’s approach for improving the quality and volume of coverage of SRHR research in the mass media focused on three major strategies:

- Creating and sustaining interest among journalists in covering SRHR research

- Building the capacity of both journalists (to understand and report SRHR research) and researchers (to share SRHR research in simple and accessible ways)

- Building and sustaining trust, enhancing positive understanding and mutually beneficial relationships between journalists and researchers

As noted earlier, our efforts in building capacity, trust and mutual relationships between journalists and researchers were informed by the partnership approach which builds social capital that involves developing high levels of co-operation, reciprocity and trust as members of the community work together for mutual social benefit [14,15].

#### Creating and sustaining interest and motivation among journalists to cover SRHR and other health research

The mass media, as already observed, are not always interested in or motivated to cover research, including SRHR research. Therefore, a key strategy for improving coverage of SRHR issues in the media should involve sparking motivation and interest in these issues. Since 2004, APHRC has forged partnerships with journalists and journalist associations and carried out various activities to motivate journalists to report on SRHR and other health issues. In 2004, APHRC signed a Memorandum of Understanding (MoU) with the Kenya Union of Journalists (KUJ) to promote media coverage of health issues in Kenya. Through this partnership, APHRC presented research findings on health inequities in a media briefing, organized a training workshop for journalists on reporting on health issues, and organized journalist visits to the Center’s field research sites in two slum settlements in Nairobi, in order to inspire and motivate them to prepare stories on health inequity issues.

In 2007 and 2008, APHRC carried out a series of activities to motivate journalists to report specifically on SRHR. The activities included organizing two journalist awards with cash prizes for good reporting, backed up by training activities to highlight key SRHR issues and sources of evidence. The aims of the awards were to raise the awareness of journalists about SRH issues, motivate them to report on these issues, enhance accurate reporting, and increase the status and prestige associated with reporting on SRHR.

APHRC’s Sexuality Journalist of the Year Award, launched in 2007, was designed to encourage informed reporting on sexuality issues in East Africa. Given that sexuality issues in Africa are often under-prioritized and therefore superficially investigated by the mainstream media, the award sought to recognize the commitment and contribution made by print media journalists on sexuality. The award for each of the participating countries (Kenya, Tanzania, and Uganda) carried a cash prize of the equivalent of US$675 for the top winners, and honorable mentions via press releases and APHRC’s newsletter for the first and second runners-up. Certificates from APHRC were presented to the top three journalists from each country, following an evaluation of their articles submitted over a six-month period. Stories submitted for the Sexuality Award covered various SRHR issues including sexual violence (rape), male circumcision, cervical cancer, family planning, stigma surrounding HIV testing, early marriages, early pregnancies, and initiation of sexual intercourse. Titles of some of the winning stories and other information on the award are captured in Additional file 2. For this award, 16 stories were submitted from Uganda and Tanzania. While Kenyan journalists published a considerable number of important and commendable stories on sexuality issues during this period (as noted through newspaper reviews by the authors), none of these were entered for the award.

The second award was the Fifth African Population Conference Competition for Journalists, organized in partnership with the Union for African Population Studies (UAPS). The award offered cash prizes (US$800 for the winner, US$400 for the first runner-up, and US$200 for the second runner-up) for the best print and electronic reporting on research presented at the conference, which was held from 10-14 December 2007 in Tanzania. To enhance the quality and quantity of media coverage of the conference, a workshop for journalists was carried out before the start of the conference. The workshop introduced the conference to journalists, discussed the key population and development issues the conference would be deliberating, and facilitated discussions between journalists and researchers. Other activities aimed at enhancing media coverage of the conference included the distribution of an information kit about the research at the conference, and making APHRC and other institutions’ staff available throughout the conference to provide advice on reporting. Additional measures taken to enhance the motivation of journalists included funding some of them to attend the conference, and building personal relationships with journalists during the conference and via subsequent email and telephone communications. The conference was attended by 74 journalists from nine African countries. We tracked down 64 stories that were reported in the media, 38 of which were submitted for this Award (Additional file 3 provides more information on this award and the winning stories).

While it was hard to attract journalists, especially Kenyan journalists, to enter the Sexuality Award, the Fifth African Population Conference Competition for Journalists received a high number of entries from various African countries, including Kenya. Broadness of population themes covered in the Conference and journalists’ level of prior exposure to these issues may have contributed to this success. Furthermore, some journalists indicated that they valued the personal contact and interaction with APHRC staff and others during and after the African Population Conference. Staff from APHRC and the Population Reference Bureau (PRB) interacted with individual journalists in the conference’s press gallery where they responded to questions, discussed interesting issues arising from the conference sessions, provided evidence for stories, and facilitated interviews with scientists and policy makers at the conference. After the conference, APHRC staff made follow-ups with journalists encouraging them to continue developing stories as well as to feel free to ask for information whenever necessary. This personal interaction enabled journalists to quickly access the information they needed for their stories and meet editorial deadlines. It also improved the quality of media stories as journalists were able to quickly clarify any issues with scientists as well as support their stories with scientific evidence. The criteria for the selection of winners were published in advance and included a detailed description of the issues. Some reporters noted that the support and documents they got motivated them to produce features on key issues at the conference as opposed to short news reports on the conference.

Poor response from journalists in entering the Sexuality Award, on the other hand, could also have been due to the fact that there was no sustained provision of information on sexuality issues, nor training workshops to point journalists to specific issues that they could cover. For instance, a Kenyan journalist noted that she was not sure what exactly she was expected to report on regarding sexuality. The need for close interaction and detailed briefing of journalists is supported by the studies by Population Council’s FRONTIERS project and Futures Group POLICY Project in Egypt and Indonesia, which found that there was an increase in coverage of reproductive health issues in the mass media within the two countries after the two continuously organized press briefings and provided background materials to journalists during a period of one to one and half years [16]. The time and effort involved in following up journalists to cover health research in the media is one of the major challenges that research organizations face. In our experience, building long-term relationship with journalists presents the best way to address this issue – when trust is built between researchers and journalists, journalists actually often become pro-active in consulting researchers for evidence and technical advice on various issues that they are covering.

The amount of cash awards and the number of winners also matter in motivating journalists to report on issues. We interviewed some Kenyan journalists to understand why they did not enter the Sexuality Award despite writing some articles on the topic during the competition period. Some of these journalists indicated that they did not enter the competition because the cash amount was low and the award provided for only one person in each country to win the cash award, while the first and second runners-up would only receive honorable mentions and certificates. Furthermore, some journalists indicated that the requirement of having them submit their best sexuality-related work was an arduous task compared to APHRC simply keeping track of their stories and selecting whichever they found to be appropriate for the competition. These sentiments were expressed during a side discussion that APHRC organized during the annual general meeting of the Media for Science, Health, Environment and Agriculture in Kenya (MESHA) in Nairobi in 2007. The Fifth African Population Conference Competition, on the other hand, offered slightly bigger cash awards (for the top winner) and, more importantly, it provided cash awards for the first three winners for each category. It is worth noting that its prizes were still low compared to similar awards in other competitions. For example, the CNN African Journalist Awards not only offer cash awards; they also provide other lucrative prizes such as a computer, a printer, and a trip to the CNN headquarters in Atlanta for the overall winner. Despite this, the cash prizes and certificates offered in APHRC’s media competitions were valued by the winning journalists. A winner of the Fifth African Population Conference Competition commented:

“It’s such a great honor to me being the first award I have won in my career and it gives me great motivation to work even harder.”

#### Building Capacity of Journalists to Use, and Researchers to Share, SRHR Research

##### Training Journalists on how to access, interpret and use research evidence

APHRC implemented a number of initiatives to strengthen the capacity of journalists to accurately interpret and report SRHR research, as well as the capacity of researchers to communicate research in comprehensible ways. One of these initiatives involved the facilitation of a journalists’ training workshop in 2004 to increase their awareness and understanding of inequities in health, including reproductive health, among urban poor and urban non-poor residents. This one-day workshop featured presentations on the major inequities in health in Kenya. Each presentation was followed by questions and discussions between journalists and researchers on the main issues arising from the research and their implications for Kenya’s development. Journalists were also given a research report containing evidence on the health inequities. As part of this capacity strengthening activity, APHRC organized a tour of the informal settlements in Nairobi, where much of its research is conducted, for the journalists. As a result of the workshop and the journalists’ visit to informal settlements, more than 20 media stories appeared in newspapers, TV and radio in the three months following the workshop.

Another training event for journalists was conducted on reporting on “Violence against Women and Girls in Africa: Health Effects and Implications”. This was part of the International Women Media Foundation workshop for journalists in Nairobi to commemorate the International Women’s Day on March 8, 2007.

##### Providing technical input in the development of TV Drama

Another initiative involved working with TV drama to communicate research on selected SRHR issues. Since November 2006, APHRC worked with the company MEDIAE to provide input on SRHR issues for their development education TV drama series, Makutano Junction, which is aired in a number of African countries. This involved providing ideas and feedback about SRH rights messages on rape, intimate partner violence, obstetric fistula, reducing stigma on homosexuality, and abortion for the development of messages, stories, scripts and information comics over a three year period. In this case, building capacity was two way. We were providing research evidence and sustained support to MEDIAE in developing SRH rights-based messages. This included holding a briefing session on SRH rights for scriptwriters. We were also learning from their skills in simplifying and framing messages for the general public. The evidence we provided to MEDIAE combined both APHRC’s research with evidence from other research organizations; therefore, part of our role was to synthesize the latest evidence available. One of our aims with communicating research evidence in this way was to tackle stigma associated with survivors of rape or violence, women affected by fistula and women who have abortions. Another aim was to spread awareness of the causes of health problems such as the risks of unsafe abortions. The final aim was to communicate information about services available and how to access them; this included, for example, highlighting post-rape and post-abortion care services both during the TV episodes and in more detail through follow-up information resources in the form of comics.

APHRC and MEDIAE put a considerable amount of time into honing messages on sensitive issues, especially in relation to abortion. We also consulted SRHR advocates in strategizing about the messages. Feedback that viewers sent to MEDIAE through SMS (short message service) showed how useful they found the shows and raised questions on various issues related to the topics. After an episode on abortion was aired in Kenya, for example, MEDIAE received 1,476 text messages from viewers, including comments on the show, questions about abortion and requests for the information comics. 1,093 viewers provided their postal address and were sent the information comics.

##### Building the capacity of researchers in engaging with the media

On building the capacity of researchers to communicate SRHR research in simple formats and how to deal with the media, APHRC organized mini-media workshops for its staff and other researchers. The aim of the workshops was to enable researchers to talk about their research in simplified language that journalists can easily understand and report about. The workshops enabled researchers to understand how the media work and the aspects of research that the media are particularly interested in. They covered important practical issues such as whom to contact, how to relate to the media, how to prepare for media interviews, how to prepare news as well as feature stories for the media, and the editorial process of selection and prioritization of stories. The workshops were co-facilitated by seasoned journalists who have had extensive experience in media houses and have interacted with researchers or covered research. Besides the short in-house mini-workshops, APHRC funded several of its researchers to attend training courses on communicating research to policymakers organized by PRB, which usually cover components on effective communication of research through the mass media.

APHRC communication staff also conducted various training activities for researchers across the continent on effective communication of research outputs, including engagement with the media. Some of these activities were organized jointly with the communication staff from the Guttmacher Institute at major international conferences held in Africa including, *Investing in Young People’s Health and Development Conference* in Abuja, Nigeria (April 27-30, 2008), World *YWCA’s International Women’s Summit* in Nairobi (July 3-11, 2007), *2^nd^ Africa Conference on Sexual Health and Rights* in Nairobi (June 19-21, 2006), and *14^th^ International Conference on HIV/AIDS and STIs in Africa* (ICASA) in Abuja (December 4-10, 2005).

APHRC researchers found the communication trainings useful in enabling them to effectively talk about their research to journalists, policymakers and the general public, as shown in the excerpts from the staff members’ workshop evaluation notes below:

“It was very informative in that I had a different perspective and appreciation of what the media have to go through as they try to sell the space or airtime. I have since drafted a research-based item that has been less scientific and more informative to non-academics with communication staff’s input … I almost feel that I can happily relate an APHRC thought in “journalistic” style …” Associate Research Scientist, APHRC.

“I found the media sessions to be very useful. Getting to know some journalists in a relaxed environment was useful to me for getting over the ‘mental hurdle’ of being ready to be interviewed by journalists. It helped increase my confidence for dealing with the media and helped me to prepare for a radio interview that I did with the Kenya Broadcasting Corporation’s Health Program on menstruation in informal settlements.” Senior Research Officer, APHRC.

The unique aspect of this two-way capacity building effort is that researchers were involved in facilitating the training of journalists, and journalists were involved in facilitating training of researchers. This mutual approach to capacity building helped to enhance capacity while bridging the communication gap between the two groups. The need to train researchers on how to talk about their research in a simplified manner had been identified by an APHRC 2007 media study.

#### Building trust and mutual relationships between researchers and journalists

From our experience working with journalists, a major barrier to coverage of SRHR and other health research in the mass media was the lack of trust and mutual relationships between researchers and journalists. Researchers did not trust journalists as they feared that journalists would sensationalize or misrepresent their research. Journalists, on the other hand, often viewed researchers as being arrogant and elitist, lacking respect for journalism as a profession and being unwilling to simplify their research for consumption by the general public.

Evidence from the APHRC media study revealed that many journalists felt that researchers were uncooperative in sharing their research [APHRC 2007 Report of the Situation Analysis Survey on Reporting of Research on Health and Population in Kenyan Media]. Reasons identified by journalists for researchers’ uncooperativeness included: lack of time, suspicion, desire to protect intellectual property, being media shy, fear that journalists will misrepresent their work, and lack of understanding of the role of the media. This study recommended the need for ‘frequent communication and meetings between journalists and scientists [to facilitate] exchange of experiences, ideas, and stories’ [ibid]. Such regular interaction would help address suspicion and lack of trust between researchers and journalists.

In response to this challenge, APHRC implemented initiatives that sought to create awareness about its work among journalists and provide forums where APHRC researchers interact with journalists on an ‘equal-level,’ with each party feeling free to share their work and experiences. These initiatives involved working with professional associations of journalists; holding regular informal forums between APHRC staff and journalists; and establishing and sustaining personal-level relationships with journalists.

APHRC explored forging sustained and mutual relationships with journalists as a way of not only providing information, but also of building trust between journalists and researchers.

##### Working through journalist associations

The partnership with KUJ in 2004 was very useful in bridging the communication and trust gaps between APHRC’s researchers and journalists, and substantially enhanced the reporting of APHRC’s research in the media. Unfortunately, this promising partnership was short-lived due to leadership squabbles that developed in KUJ during most of 2005 and 2006. APHRC joined the Media for Environment, Science, Health and Agriculture in Kenya (MESHA) as a corporate member in 2007. This membership helped facilitate greater participation of MESHA members in APHRC’s dissemination activities. Furthermore, the MESHA secretariat served as a platform for APHRC to share its research with specific journalists known to prepare good quality science stories, and this boosted media coverage of research. We also used MESHA’s general meetings to share our research through brief presentations and talks by researchers followed by discussions with journalists; we specifically participated in the 2007 and 2009 MESHA general meetings.

##### Holding informal forums and establishing personal links with journalists

APHRC forged personal relationships between individual journalists and APHRC staff. This was done through the organization of regular informal forums, mainly luncheons held at APHRC offices. These sessions facilitated the development of better relationships between APHRC researchers and journalists as well as increased knowledge among journalists of APHRC’s research, and knowledge among researchers on the work of journalists and the challenges they struggled with when reporting on research. In addition to these forums, APHRC’s communication staff embarked on a mission to develop personal relationships with individual journalists (including science editors, science writers, and science correspondents), and linked the journalists with research staff. In order to sustain these relationships the communication team at APHRC initiated a strategy of having one-on-one and group informal meetings with journalists to facilitate exchange of information and ideas on possible stories.

Journalists who participated in APHRC’s training workshops and meetings with researchers found these useful in reducing the barrier between them and researchers and enabling them to develop better working relationships with researchers. A freelance science journalist, who writes for the *Daily Nation*, the leading daily newspaper in Kenya and who attended two of the meetings with researchers noted that:

“The meetings were very useful, in the sense that they helped dispel the notion that journalists and scientists do not usually get along very well. … Journalists got to know more about the researchers on an informal level, which I think, went a long way to cultivate trust between the two professionals.” Freelance Science Journalist, *Daily Nation*, Kenya.

### What was the impact of our media engagement?

Measuring the impact of media engagement and other research communication strategies is a big challenge for a number of reasons. For instance, such strategies may require a certain amount of time to influence how journalists value and use research in their work. One key objective of APHRC’s media engagement strategy has been to improve both the volume and quality of reporting on health and SRH issues. While it may be reasonably easy to track changes in the volume of reporting, assessing the quality of reporting requires more detailed content analysis of the stories and documentaries that journalists put together. This level of analysis necessarily requires more financial and technical resources as well. The absence of an independent institution geared toward building the capacity of journalists to apply research evidence in media reporting for its own sake (as opposed to for the purposes of promoting specific research), results in most media engagement activities being carried out by institutions whose main interest is to promote the coverage of their own work in the media. In this case, monitoring of changes in reporting of specific issues fails to capture the overall impact of the intervention since journalists apply these skills in their work in other areas, and they also cover work done by other research institutions. As the narratives above show, APHRC’s media engagement strategy was focused on strengthening capacities of journalists and visibility of specific issues (e.g. sexuality awards), as well as on promoting the application of research in general by the media (Fifth African Population Conference awards).

APHRC’s communications team mostly tracks coverage of stories that directly relate to the institution’s work (with the exception of coverage of research presented in its conferences or awards that are issued), yet the strategy goes beyond APHRC’s staff and research work. The media engagement strategies managed to sustain a high level of coverage of APHRC’s work (including SRHR issues) in the media over the five-year period. In 2004 there were 24 appearances, compared to only 4 in 2005 and 18 in 2007. Coverage was particularly low in 2005 because the Center did little media engagement following the stalled partnership with KUJ. Nonetheless, the numbers picked up again in 2006 when the Center started implementing many of the strategies described above.

There were 75, 24, and 85 media appearances in 2007, 2008, and 2009, respectively. The exceptionally high levels of reporting in 2007 and 2009 were mostly due to the media engagement activities that were linked to two major conferences that APHRC co-organized. The numbers in these years also included research presented at the conferences by the broader research community, and not only APHRC research. In 2007, the sexuality award and the award linked to the Fifth African Population Conference played a key role in ensuring such a high level of media coverage. The high number in 2009 was mainly due to media activities linked to the International Conference on Urban Health and the informal meetings held between researchers and journalists during this period (although the full impact of this activity will be assessable in future years). We also implemented two media training sessions for APHRC researchers in the course of 2009.

Regarding SRHR, the media made it possible for us to use research to raise the visibility of, and challenge various stakeholders to address, the stigma and silence that surround many SRHR issues in sub-Saharan Africa and in turn hinder people from accessing information and, consequently, services. For instance, the public’s requests for information on SRHR issues (specifically, abortion and rape) covered by the TV drama Makutano Junction points to how relevant and useful the media was in communicating SRHR information that is not readily available to the public due to stigmatization of SRHR issues and silence. The fact that both the journalists and researchers felt that their eyes were opened to the importance of the two groups working together, and to ways of effectively doing so, show that the strategies have the potential to enhance both the quality and volume of evidence-based coverage of SRHR and other health issues in the media. A big challenge remains in regard to sustainability over time as one cannot expect to see long-term visibility of sensitive issues like sexuality through a few journalist competitions or seminars.

Finally, it is much harder to attribute specific media engagement strategies to the ultimate audiences that researchers often seek to reach. As noted above, apart from informing the public about research in health, the media engagement strategy provides an additional route for reaching out to leaders, policy makers, program designers, development partners, and other stakeholders, who are major consumers of media products. In APHRC’s case, a whole range of other communication and policy engagement strategies are used to influence policy and practice, including one-on-one meetings and dissemination workshops, scientific publications, policy briefs, participation in government technical working groups, and partnership with policy makers in designing and implementing research. For example, APHRC’s research has made a substantial contribution in drawing national (in Kenya), regional, and global attention to the plight of the urban poor. APHRC provided some of the evidence that resulted in a major investment by the Bill and Melinda Gates Foundation in addressing the sexual and reproductive health needs of the urban poor in Africa and Asia. The government of Kenya is also paying much greater attention to the service needs of slum residents in Kenya than was the case in 1999 when APHRC first started working in these communities. Clearly, policy and practice changes of this nature do not happen overnight. It took a sustained commitment on the part of APHRC to generate policy-oriented research outputs and communicate them to policy makers and other end-users through a series of strategies, including media engagement.

## Conclusion

The media can play a valuable role in communicating important research findings and raising the profile of overlooked and contentious public health issues, such as SRHR, to the public, including political leaders, policy makers, and key stakeholders. Reporting of these issues is weak due to lack of interest and capacity in accessing, interpreting and using evidence by journalists, as well as lack of capacity to communicate research findings effectively by researchers. Addressing these issues is a worthwhile, yet extremely challenging endeavor that needs recourses, commitment, and patience on both sides. This paper documents APHRC’s efforts in addressing these challenges between 2004 and 2009. The Center’s media strategy evolved over the years, moving beyond conventional ways of communicating research through the media (such as circulating news releases and writing newspaper stories), to adopting interactive approaches informed by the partnership approach and which aim to motivate journalists to report SRHR research, build capacity of both journalists and researchers, and develop trust and good working relationships between researchers and journalists. Based on our experiences and reflections documented in this paper, we highlight the following as key ingredients of successful media engagement strategies:

• For **media awards of excellence** to succeed in promoting quality coverage of SRHR research, they need to be accompanied with training of journalists, provision of detailed information on the subject matter and what is expected of them, and provide continuous encouragement and support. It would also be helpful to involve journalists in designing the awards, including their suggestions on what kind of awards and incentives would appeal to them.

• **Capacity building efforts** to improve the quality and quantity of mass media coverage of SRHR research need to target both journalists and researchers. Such efforts should go beyond one-time activities or events to involve continuous interaction, mentoring, and follow-up with participating groups.

• Efforts to **improve mutuality and reduce mistrust** between journalists and researchers should include establishing both formal and informal relationships between journalists and researchers. Opportunities for regular interaction and joint skills building can help to promote mutual understanding, respect and trust between the two groups.

• It is important to set up proper **monitoring and evaluation** tools to enable assessment and documentation of the impact of the media engagement in promoting communication and use of research evidence by the general public, leaders, policy makers, and key stakeholders in SRHR. The monitoring and evaluation tools should enable assessment of the impact of individual intervention strategies as well as comprehensive packages on both volume and quality of reporting. The tools should also enable assessment of efforts by individual institutions as well as the collective impact of strategies by various institutions on a given issue.

• For research communication strategies of research organizations to be effective, there is need for **close collaboration between communication specialists and researchers** in designing and implementing the strategies.

## List of abbreviations

APHRC: African Population and Health Research Center; CNN: Cable News Network; ICASA: International Conference on HIV/AIDS and STIs in Africa; KUJ: Kenya Union of Journalists; MEDIAE: The Media for Education and Development Company; MESHA: Media for Environment, Science, Health and Agriculture in Kenya; MoU: Memorandum of Understanding; SRH: Sexual and Reproductive Health; SRHR: Sexual and Reproductive Health and Rights; UNFPA: United Nations Population Fund; YWCA: Young Women’s Christian Association

## Competing interests, author contributions and declarations

This article critically reflects on a research project in which the authors have been involved. All the four authors participated in the conception of the paper. RNO led in drafting the paper. CU, JC and EMZ drafted some sections of the paper. All the authors reviewed and contributed to the revising of the paper, and they have seen and approved the final version. The authors declare that they have no competing interests.

## Authors’ information

RNO served as the Communications Officer at APHRC from 2004-2008 and Communications Manager in 2009. She therefore, played a key role in formulating and implementing all the activities discussed in this paper. CU served as a post-doctoral fellow from 2004-2006, and an Associate Research Scientist from 2006-2009 at APHRC. Her research focus was on sexuality and sexual and reproductive health and rights issues in sub-Saharan Africa, and she therefore actively participated in the research communications activities discussed in this paper. EMZ served as the Director of Research and Deputy Director at APHRC from 2001-2009. Part of his work in this position included overseeing APHRC’s research communications strategy and therefore worked very closely with APHRC’s communications team in formulating and implementing the Center’s research communications strategy. JC served as a Research Officer at APHRC between 2005-2009 and her research focused on sexual and reproductive health issues in sub-Saharan Africa. JC therefore worked closely with APHRC’s communications team in designing and implementing research communication related to sexual and reproductive health research.

## Supplementary Material

Additional file 1 The African Population and Health Research Center (APHRC) in Brief Click here for file

Additional file 2 The APHRC Sexuality Journalist of the Year Award Click here for file

Additional file 3 The Fifth African Population Conference Competition for Journalists Click here for file
