# Supplementary material for: Engaging media in communicating research on sexual and reproductive health and rights in sub-Saharan Africa: experiences and lessons learned
Source: Health Res Policy Syst. 2011 Jun 16;9(Suppl 1):S7. doi: 10.1186/1478-4505-9-S1-S7 (PMC3121138; doi:10.1186/1478-4505-9-S1-S7)
Supplement: Additional file 1 — The African Population and Health Research Center (APHRC) in Brief [file 1478-4505-9-S1-S7-S1.pdf]

Table 1: The African Population and Health Research Center (APHRC) in Brief

APHRC is a non-profit, non-governmental, international organization that is committed to conducting policy-relevant research on population, health and development issues in sub-Saharan Africa. The Center also facilitates the use of research evidence in policy and practice, in addition to strengthening the research capacity of African scholars and institutions to enhance skills in generating credible scientific evidence. APHRC has three core priorities:

- Contribution to science through high impact research projects and publications
- Inform policy decisions with research evidence
- Strengthen research capacity in sub-Saharan Africa

APHRC's vision is to be a global center of excellence, consistently delivering sound scientific evidence for policy and action. APHRC's mission is to promote the wellbeing of Africans through policy-relevant research on population and health. Website: www.aphrc.org
