# Supplementary material for: Engaging media in communicating research on sexual and reproductive health and rights in sub-Saharan Africa: experiences and lessons learned
Source: Health Res Policy Syst. 2011 Jun 16;9(Suppl 1):S7. doi: 10.1186/1478-4505-9-S1-S7 (PMC3121138; doi:10.1186/1478-4505-9-S1-S7)
Supplement: Additional file 2 — The APHRC Sexuality Journalist of the Year Award [file 1478-4505-9-S1-S7-S2.pdf]

Table 2: The APHRC Sexuality Journalist of the Year Award

The APHRC Sexuality Journalism Award was launched in March 2007, with funding from the Ford Foundation, and ran for six months to end in November the same year. The award was restricted to Kenya, Tanzania and Uganda print journalists, aimed to recognize the contribution of print journalists to the public's understanding of sexuality and its linkages to socio-economic development concerns in the sub-region. The award was announced through various activities including:

- A news release circulated via email to mainstream media houses (news-desks), journalist networks and associations, and individual journalists in the three countries;
- In Tanzania at a public forum on 'Sexuality, law and culture in Africa' in March 2007 organized by APHRC and the University of Dar-es-Salaam;
- In Kenya in April 2007 at a public forum on 'Sexuality and the Academy' organized by APHRC and Moi University in Eldoret;
- APHRC's January-April 2007 newsletter and APHRC website.

Through these announcements, journalists were referred to the APHRC website where they would download entry forms and other information on the award. Reminders on the award were circulated via email every month throughout the six-month period, between March and November 2007. APHRC communications staff also publicized the award in two journalist forums in Kenya during this period. In one of these forums, it emerged that some journalists did not quite understand the meaning of sexuality and the kind of stories they were expected to cover. So as a response, definitions of sexuality were also circulated to journalists via email.

In total, the award attracted 16 entries from Tanzania and Uganda; no entries were submitted by Kenyan journalists. The stories were assessed based on the criteria below by 6 judges, comprising two APHRC communication staff, two APHRC sexuality researchers, and two additional APHRC researchers working in the area of sexual and reproductive health, although not specifically on sexuality.

- Degree of consistency in reporting
- Demonstration of grasp of sexuality issues
- Balanced coverage of sexuality issues
- Creativity and innovativeness
- Quality of entries
- Proven/potential impact

The winning stories included:

- 'Cut' fever hits Uganda (story on male circumcision) by Bamutaraki Musinguzi, Uganda
- Silent Killer (story on cervical cancer) by Bamutaraki Musinguzi, Uganda
- Babies before school: the story of Kisarawe by Eric Kabendera, Tanzania
- Family planning and huge tasks ahead by Eric Kabendera, Tanzania
- The dilemma of rape victims by Kakaire Ayub Kirunda, Uganda
- The push for an anti-FGM law by Kakaire Ayub Kirunda, Uganda
