# Supplementary material for: Engaging media in communicating research on sexual and reproductive health and rights in sub-Saharan Africa: experiences and lessons learned
Source: Health Res Policy Syst. 2011 Jun 16;9(Suppl 1):S7. doi: 10.1186/1478-4505-9-S1-S7 (PMC3121138; doi:10.1186/1478-4505-9-S1-S7)
Supplement: Additional file 3 — The Fifth African Population Conference Competition for Journalists [file 1478-4505-9-S1-S7-S3.pdf]

Table 3: The Fifth African Population Conference Competition for Journalists

This award was implemented as part of the 2007 Fifth African Population Conference, organized by the African Union for Population Studies (UAPS), the Government of the Republic of Tanzania, and APHRC. The award was funded through the Realizing Rights research program consortium, supported by the UK Department for International Develop (DFID). The purpose of the award was to motivate journalists participating in the conference to report on issues discussed at the conference in order to raise the profile of these issues at national and international levels. Seventy-four (74) journalists and photographers from Burkina Faso, Burundi, Cameroon, Kenya, Malawi, Niger, Rwanda, Tanzania, and Uganda participated in the conference. The award carried cash prizes of US$800, 600, and 400 for the Winner, 1st and 2nd runners up, respectively, in print, TV and radio media categories. The award was launched at the conference and all journalists participating in the conference were informed about the award and its criteria. To provide journalists time to research and develop in-depth stories, the award ran from December 10-February 29, 2008. After the conference, APHRC made regular email follow-ups with the journalists for updates on the stories they were working on and to encourage them to submit stories for the competition. In total, 38 stories were submitted for the competition. These were assessed by sixAnglophone, Francophone and Swahili-speaking judges, including two APHRC communication staff, two APHRC researchers, one communication experts from Kenya's National Coordinating Agency for Population and Development and a member of the Kenyan media association MESHA. The criteria for assessment were:

- Accuracy of reporting
- Use of research evidence from conference proceedings
- Relevant to at least one conference theme
- Entries had to refer to the conference by name to be eligible for consideration

Some of the comments from judges who assessed story entries for the Fifth African Population Conference Competition for Journalists

Comments on winning articles	***Comments on non-winning articles***
"Good story. Balances views from different sources – researchers, policymakers, etc"	"Good story. Puts a face to research. But fails to even state Kenya's fertility rate yet it's about poverty & fertility."
"Good news piece. Provides some level of depth. Cites the president and TZ [Tanzanian] minister."	"Story lacks important details like countries where study was done, key challenges facing young people as far as RH [Reproductive Health] is concerned, etc."
"Good story. Good depth and very well written."	"Good story. Mentions conference, but reads like the journalist did not speak to anyone at conference nor used evidence from the conference."

NB: These comments are for different stories submitted and are listed here as examples of some of the assessments that were made about entries to the competition.

The winning stories included:

- 'Cheap, easy drug to save mothers from bleeding to death' by Alice Emasu and Irene Nabusoba, *New Vision*, Uganda.
- 'Démographie africaine: Images et mirages autour des objectifs du millénaire' by Xavier Messe, *Quotidienmutations*, Cameroon
- 'Where Marriage Doesn't Count' by Redemptor Atieno, *Daily Nation*, Kenya.
- 'Vital Health services still a far cry in Africa' by Ayoki Onyango, *Saturday Standard*, Kenya.
- 'Population Afrique: Que font les gouvernements pour arrêter la fuite des cerveaux?' by Aimable Twahirwa, *Inter Press Service*.
- 'Fertility in Africa' by Richard Chacha, *NTV*, Kenya.
- 'Poverty and Fertility Rate in Kenya' by Lydia Kusia, *Family FM*, Kenya.
